# Nano-scale characterisation of sheared β” precipitates in a deformed Al-Mg-Si alloy

**DOI:** 10.1038/s41598-019-53772-4

**Published:** 2019-11-25

**Authors:** Emil Christiansen, Calin Daniel Marioara, Bjørn Holmedal, Odd Sture Hopperstad, Randi Holmestad

**Affiliations:** 1 Centre for Advanced Structural Analysis (CASA), NTNU - Norwegian University of Science and Technology, Trondheim, N-7491 Norway; 2 Department of Physics, Faculty of Natural Sciences, NTNU, Trondheim, N-7491 Norway; 3 Materials and Nanotechnology, SINTEF Industry, Trondheim, N-7465 Norway; 4 Department of Materials Science and Engineering, Faculty of Natural Sciences, NTNU, Trondheim, N-7491 Norway; 5 Department of Structural Engineering, Faculty of Engineering, NTNU, Trondheim, N-7491 Norway

**Keywords:** Metals and alloys, Imaging techniques

## Abstract

This paper compares the nano-scale structure of β” precipitates in a peak-aged Al-Mg-Si alloy before and after deformation. Three complementary advanced transmission electron microscopy techniques are used to reveal the structures and elucidate the interaction between dislocations and β” precipitates. We show that the needle-like and semi-coherent β” precipitates are sheared several times on different planes by dislocations during deformation, with no indications that they are bypassed or looped. Our results show that dislocations cut through precipitates and leave behind planar defects lying on planes inclined to 〈100〉 directions inside the precipitates. The results also indicate that precipitates are sheared in single steps, and the implication of this observation is discussed in terms of slip behaviour.

## Introduction

Age-hardenable aluminium alloys, such as the Al-Mg-Si alloys, have a high strength to weight ratio and offer valuable alternatives to e.g. steels in a number of industrial applications. However, the mechanical properties of these alloys can be challenging to predict, as they depend on numerous parameters. Therefore, numerical models are valuable tools for both alloy development and product design in for example metallurgical, automotive, and aerospace industries^1–4^. In this respect, knowledge and understanding of the underlying physical processes responsible for the mechanical properties are essential. One of the most important physical processes is the interaction between gliding dislocations and nanoscale metastable precipitates in the material, as this interaction has a large impact on strength, work hardening, and fracture^5,6^. Because the dislocation-precipitate interactions occur on the nanoscale and at very short time intervals, they are not easily accessible for direct study, but can be indirectly studied by comparing the atomic structure of precipitates before and after deformation.

Precipitates in age-hardenable aluminium alloys are formed toward the end of the heat-treatment process and serve as obstacles to moving dislocations. They usually start out as small and coherent phases embedded in the aluminium matrix early in the ageing procedure. With increasing ageing time, they grow and change both in structure, composition, and shape. To predict the strength and work hardening of age-hardenable aluminium alloys, it is necessary to know both the phase, size, shape, and number density of precipitates as well as the individual dislocation-precipitate interactions for the various precipitate phases. There are two main types of precipitate-dislocation interactions: bypassing and shearing. Whether a particular precipitate is bypassed or sheared depends on the phase, size, and shape of the precipitate.

Dislocations bypass non-shearable precipitates during deformation by looping mechanisms, which result in various dislocation structures around the precipitate^7^. For example, the Orowan mechanism leaves precipitates encircled by dislocation loops that increase the dislocation density and contribute to the initial work hardening of the material.

When shearable precipitates are cut by dislocations, steps will form on the precipitate-matrix interface and various defects inside the precipitate structure can form^8^. The shearing mechanism is directly linked to the structure of the precipitate, and different precipitate phases will behave differently during deformation. For instance, Deschamps *et al*.^9^ observed that both of the lath-like *δ*′ and T_1_ precipitates in an Al-Cu-Li alloy were shearable, but the T_1_ precipitates were sheared in single steps only. Slip was localised in the *δ*′ strengthened alloy, but not in the T_1_ strengthened alloy. Hence, even though precipitates in both cases were shearable, the mechanism for shearing was different and this affected the mechanical properties. In other words, complex shearing mechanisms must be understood for each precipitate phase present in an alloy.

The precipitates in peak-aged Al-Mg-Si alloys are usually of the β″ phase, which is semi-coherent with the matrix and appear as needles oriented along 〈001〉_Al_^10,11^. *In situ* investigations performed on an Al-Mg-Si-Cu alloy, containing both needle-like β″ precipitates and lath-like L precipitates, suggest that both shearing and bypassing of precipitates occur at room temperature, and that increasing temperature facilitates cross-slip and bypassing^12,13^. However, whether the lath-like or needle-like precipitates are sheared was not determined. Donnadieu *et al*.^14^ measured the strain fields around the precipitates and performed simulations of the dislocation-precipitate interactions. Their results suggest that the lath-like precipitates were sheared, and the rod-like precipitates were looped. However, the *in situ* study by Misumi *et al*.^15^ shows that β″ precipitates are shearable, indicating that the strain field simulations by Donnadieu *et al*. might have some shortcomings. Ryen *et al*.^16^ showed that after large deformations, the needle-axis of β″ precipitates changed, indicating that they were uniformly sheared by dislocations. Furthermore, Poole *et al*.^17^ showed indirectly that β″ precipitates are sheared as well, through slip-line and work hardening investigations. Hence, there is strong evidence that β″ precipitates are shearable. However, despite these studies, the internal structure of β″ precipitates after deformation is still unknown. Studies of the internal structure of precipitates in plastically deformed materials could provide detailed information on the interaction between dislocations and precipitates that would be useful for models of strength, work hardening and fracture of these industrially important alloys.

The atomic structure of β″ precipitates is characterised by stackings of a specific basic unit usually referred to as a β″ “eye”^18^. The most common arrangement of eyes produces a monoclinic unit cell (space group C2/m) with *a* = 1.516 nm, *b* = 0.405 nm, *c* = 0.674 nm, and *β* = 105.3°, and composition Mg_5_Si_4_Al_2_, but less common stacking variations produce other unit cells^10,18,19^. The orientation relationship between the unit cell of the most common β″ and the matrix is ${(001)}_{{\rm{Al}}}\Vert (010)$_β″_, ${[\bar{3}10]}_{{\rm{Al}}}\Vert [001]$_β″_, and ${[230]}_{{\rm{Al}}}\Vert [100]$_β″_^10,19^.

Shearing of β″ precipitates by dislocations is a complex problem. Because the internal structure of this phase is not compatible with the matrix, the slip systems of the two phases do not match. Hence, β″ eyes cannot be shifted by a matrix Burgers vector and remain well-ordered with respect to unit cells above and below the shearing plane. There are therefore two options. Either the atoms in the shearing plane must reorganise and form a planar defect (referred to as incompatible shearing), or the eyes must shift by a compatible shift (referred to as compatible shearing). The planar defect in the former case disrupts the crystal structure of the precipitate close to the shearing plane, while the precipitate shearing and matrix deformation in the latter case will be incompatible. This incompatibility, in turn, must be accommodated by elastic strain fields in the matrix or defects at the interface between the precipitate and the matrix. The concept of compatible vs incompatible shearing is introduced to distinguish incompatible shearing with disruption of the internal structure of the precipitate from compatible shearing with disruption of the surroundings of the precipitate instead. In both cases, a step is introduced on the precipitate, but compatible shearing will also likely generate additional defects on the step. For instance, compatible shearing might lead to a residual dislocation loop on the step, with Burgers vector equal to the difference between the precipitate and matrix Burgers vectors. Based on investigations of the structure after shearing, it is thus possible to say which process has occurred, and possibly what plane the precipitate was sheared on. This will be important information, especially when considering multiple shearing events and the evolution of the precipitate strength during deformation.

Ardell^8^ describes five different strengthening contributions for shearable precipitates: *chemical strengthening* due to steps on the precipitate-matrix interface; *stacking fault strengthening* due to difference in stacking fault energies in the two phases; *modulus hardening* due to difference in elastic modulus of the two phases; *coherency strengthening* from the strain field surrounding the precipitates; and *order strengthening* due to formation of anti-phase boundaries on the shear plane within the precipitate. In the case of β″ precipitates, it seems reasonable that the local order strengthening contribution in the shearing plane will be the one most affected by shearing if β″ precipitates deform by incompatible shearing. Conversely, if β″ precipitates deform by compatible shearing instead, coherency and elastic modulus strengthening will likely be the ones most affected. Both compatible and incompatible shearing will result in a step on the precipitate, and the associated energy of this step will result in chemical strengthening. In the former case however, where the precipitate is sheared by a precipitate Burgers vector, a residual dislocation will be present at the step and likely increase the chemical strengthening contribution. Hence, determining if planar defects form in β″ precipitates after deformation will provide valuable information regarding the strength evolution of these precipitates during deformation. In addition, because dislocations in Al-Mg-Si alloys may cross-slip and change their glide plane to {001}_Al_ at room temperature^20^, it will also be important to determine which planes the possible planar defects lie on. With this in mind, there are two important questions that should be addressed: on which planes are β″ precipitates sheared by dislocations, and will this shearing of the precipitates introduce interfacial or planar defects?

To address these questions, we have studied a lean Al-Mg-Si alloy (Mg + Si less than 1 at.%) in peak hardness condition by several advanced transmission electron microscopy (TEM) techniques before and after bulk plastic deformation in compression to 5%, 10%, and 20% engineering strain. A directly correlated study where the same precipitates could be observed before and after deformation, would have been preferable. However, such studies require deformation of electron transparent specimens where dislocation sources and motion do not necessarily resemble bulk behaviour. Instead, by investigating several precipitates in different specimens after different global strain, it is possible to conclude on general changes to their morphology and eventual crystal defects. As such, this *post mortem* study aims to answer questions about the precipitate shearing process as close to the bulk configuration as possible. This has been done in two ways. Firstly, precipitates with their longitudinal axis aligned with the electron beam (i.e. oriented out-of-plane) have been studied in high-resolution TEM (HRTEM) and high-angle annular dark field (HAADF) scanning TEM (STEM). Secondly, precipitates with their longitudinal axis aligned perpendicular to the electron beam (i.e. oriented in-plane) were investigated by scanning precession electron diffraction (SPED) and non-negative matrix factorisation (NMF) machine learning^21–26^. These advanced techniques depend on complex electron interactions within the material and are succinctly described in the following.

HRTEM is a technique where the projected crystal structure is imaged through complex electron interference processes^27^. The resulting exit wave will be thickness dependent and advanced techniques are needed to directly infer the projected electrostatic potential^28^. Nevertheless, the interference between electrons will depend on the crystal periodicity, as this reflects the periodicity of the electrostatic potential in the specimen. Hence, HRTEM images will exhibit the same periodicity as that of the projected crystal lattice even for a relatively thick crystal. In the present work, HRTEM is used to study the periodicity of the projected crystal structure of precipitates and especially how the periodicity change after deformation. If precipitates contain planar defects (incompatible shearing), the projected lattice should become blurred. If the shearing is compatible with the precipitate structure, the projected crystal lattice should be preserved after deformation.

In atomically resolved HAADF STEM, a small and converged electron probe is scanned across a specimen oriented in a zone axis, and a Z-contrast image is formed by collecting electrons scattered to high angles^29,30^. The electron beam channels along the atomic columns and scatters to high angles with a probability that increases with the mass of the scattering atoms. Hence, intensities in each pixel in the resulting image scale with the average Z-number. HAADF STEM images are formed “sequentially” as the beam propagates through the specimen, and this leads to challenges when interpreting images of thick specimens, especially if there are structural variations along the beam direction. For thick specimens, the channelling process can become quite complex and prevent direct quantitative interpretation of the image^30^. Still, HAADF STEM is most sensitive to the structure of the specimen close to the entrance surface and less sensitive to the structure close to the exit surface. Defects close to the entrance surface will therefore influence the images more than defects close to the exit surface of the specimen. However, exactly how different defects and interfaces affect the channelling is difficult to predict without also performing STEM simulations. The main application of the HAADF STEM technique in the current paper is to verify the atomic structure of the precipitates after deformation. In addition, this technique will also provide valuable information for future modelling studies. As a final point, it should be mentioned that the scanning of the electron probe introduces noise that complicates the interpretation of the data. The noise can be reduced by acquiring stacks of several short-exposure images of the same area and then applying various rigid and non-rigid image registration algorithms to the data. *Smart Align* is a tool for performing such registrations, and all STEM images shown in this work have been smart-aligned^31^. However, conventional STEM images have also been acquired and used to validate the smart-aligned images.

SPED is another scanning diffraction technique, but in this case, the electron beam is precessed about the optical axis to average out dynamical diffraction effects, although some of these effects will still be present^22,24^. Unlike HAADF STEM, where all the electrons scattered to higher angles are collected for each pixel, SPED collects a complete 2D precession electron diffraction pattern for each scan pixel. The resulting dataset therefore has four dimensions: two spatial dimensions (*x*, *y*, image), and two reciprocal space dimensions (*k*_*x*_, *k*_*y*_, diffraction pattern) that contain crystallographic information. The dataset can be reduced by post-processing by creating virtual images. These images are 2D spatial maps of the intensity of certain regions in the diffraction patterns. In the case of a virtual bright field (VBF) image, the intensity within a region containing the direct beam is integrated for each diffraction pattern, and assigned to corresponding scan pixels. A VBF image is therefore comparable to a conventional TEM bright field image, but with reduced dynamical effects. Intensities within annular regions can be chosen to create virtual annular dark field (VADF) images. These virtual images provide a compact way of visualising the spatial variation of certain diffraction conditions, without the complexity introduced by dynamical effects as in conventional TEM imaging.

An alternative to creating virtual images is to apply machine learning algorithms. In this work, the NMF algorithm is used to learn the individual parts that make up different diffraction patterns^21^. NMF factorises each SPED dataset into a set of *N* factors or basis patterns with corresponding loadings or weights. The factors have the same dimensions as the diffraction patterns (*k*_*x*_, *k*_*y*_), while the loadings have the same dimension as the SPED scan region (*x*, *y*). If the diffraction patters are of size *n* = *k*_*x*_ × *k*_*y*_ and the number of scan pixels are *m* = *x* × *y*, NMF decomposes the 4D dataset of size *n* × *m* into a more manageable dataset of size *N*(*n* + *m*). The number of factors and loadings, *N*, is set by the user, and should be large enough to encompass most of the relevant signal contributions in order to avoid too strong mixing of signals. An NMF factor may be interpreted as a distinct contribution present in some of the diffraction patterns, while its corresponding loading may be interpreted as a map of how important that contribution is at any given scan position. For instance, the NMF factor that corresponds to scattering from the aluminium matrix will be strong everywhere and have a rather uniform loading map. However, an NMF factor that corresponds to reflections from a different phase, e.g. the β″ phase, will have a loading map that is weak except where the beam intersected a precipitate. It is very important to keep in mind that the NMF results are output from a machine learning algorithm and represents a statistical decomposition performed under certain constraints (in this case, a non-negativity constrain), and while qualitative observations and comparisons can be made relatively safely, quantitative comparisons between NMF results from different datasets should be avoided. However, the benefit of NMF is that the algorithm is capable of extracting intensities that are very weak in individual diffraction patterns but are statistically important in the dataset. In this work, NMF factors corresponding to in-plane precipitates have been manually identified, and their loading maps are used to highlight in-plane precipitates in VBF images. In addition, VADF images sensitive to precipitate cross-section scattering are also formed in order to separate these cross-sections from other features in both the VBF images and the NMF loading maps. This is necessary because the NMF is not completely capable of separating the relatively sparse scattering of in-plane needles from the dense scattering of precipitate cross-sections. Hence, while the HRTEM and HAADF STEM techniques provide information about the precipitate structures in cross-section, SPED can be used to investigate the precipitate structure along the length of the needle-shaped precipitate.

## Results

### Initial study

**Figure 1:**
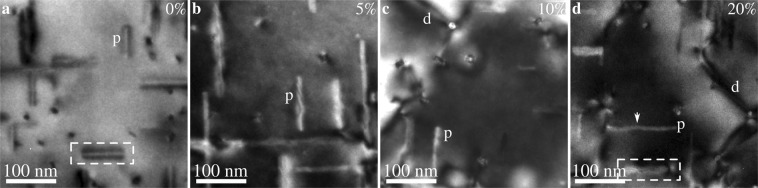
TEM [001]_Al_ zone axis bright field images of the alloy in (**a**) the undeformed condition and after (**b**) 5%, (**c**) 10%, and (**d**) 20% compressive engineering strain. Some precipitates labelled “p” have been marked, as well as dislocations “d” in some cases. The arrow in (**d**) shows a bend in one of the precipitates. Imaged areas are approximately the same as those studied using SPED. The precipitates in (**a**) and (**d**) marked by dashed boxes are studied in greater detail in Fig. 5.

An initial conventional TEM bright field study established the need for more advanced characterisation techniques. As Fig. 1 shows, the bright field contrast in deformed alloys is dominated by dislocations, making it nearly impossible to study the precipitates. Nevertheless, it is clear that the strain fields around precipitates, seen as dark regions along the needle lengths in Fig. 1a, seem to be suppressed or to disappear completely in deformed specimens. This suppression can either be due to a cancellation between the strain fields of precipitates and dislocations or because the coherency of the precipitates is lost. The latter indicates the presence of interfacial defects or internal changes inside the precipitate structure. In some cases, small disturbances in contrast (see Fig. 1b) or slight bends (see Fig. 1d) can be discerned as well.

### Study of precipitate cross-sections

**Figure 2:**
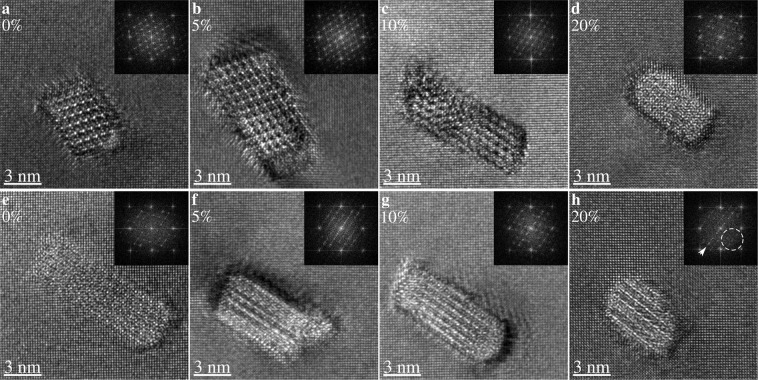
Representative [001]_Al_ zone axis HRTEM images of precipitate cross-sections in undeformed condition (**a**,**e**), and after 5% (**b**,**f**), 10% (**c**,**g**), and 20% (**d**,**h**) compressive engineering strain. The global compression axis lies in-plane. Power spectra of fast Fourier transforms of each image are inset in top-right corners. The top row (**a**–**d**) is representative of precipitates with the sharpest outline and sharpest lattice, while the bottom row (**e**–**h**) is representative of precipitates with the most blurred lattices. In (**h**), the missing precipitate frequencies are marked in the fast Fourier transform spectrum by a dashed circle, and the remaining frequencies are marked by an arrow.

Figure 2 presents HRTEM images of precipitate cross-sections reflecting the variation in sharpness of the precipitate projected crystal lattice in each condition. Precipitates in the undeformed state usually produce HRTEM images where the crystal lattice of the β″ precipitate is visible, but a few precipitates appear blurred or with reduced resolution or contrast. This variation is natural, as some precipitates do not pass through the entire thin foil and overlap with the matrix, destroying the coherency of the exit wave. With increasing deformation, precipitate cross-sections appear more and more blurred. This indicates that the projected crystal lattice of precipitates after deformation is disturbed. After 20% compression, all precipitate cross-sections appear blurred. In addition, the blurring is not uniform, and the periodicity of the crystal lattice of the β″ precipitate along one 〈110〉_Al_ is usually preserved. This is most clearly seen by the inset power spectrum in Fig. 2h. Hence, deformation appears to smear out the projected crystal lattice of the precipitates, except in one particular direction. It should be mentioned that the projected crystal lattice of precipitates in the compressed specimens is often too severely blurred to identify the crystal structure, and some of the imaged precipitates may not be β″ precipitates but have other structures. However, most of the imaged precipitates should be β″ precipitates, as this is the predominant phase in the bulk microstructure as confirmed by the HRTEM images in the undeformed condition. The blurring gives precipitate cross-sections in the deformed specimens a more roundish shape and slightly increased cross-sectional area. Measurements indicate that the mean cross-sectional areas of precipitates are 19 nm^2^ and 24 nm^2^ in undeformed and 20% compressed specimens, respectively (see Supplementary Fig. S1 for histograms).

**Figure 3:**
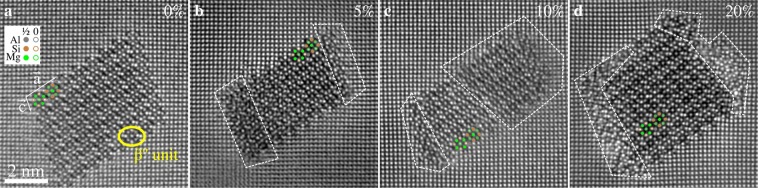
Smart-aligned and filtered [001]_Al_ zone axis HAADF STEM images of precipitates in (**a**) undeformed, and after (**b**) 5%, (**c**) 10%, and (**d**) 20% compressive engineering strain. An overlay of the β″ precipitate structure is also included, based on Wenner *et al*.^38^. The relative position of the overlaid atoms in the out-of-plane direction is indicated by full and empty circles, referring to 1/2 · *b*_β″_ and 0 · *b*_β″_, respectively. A β″ unit referred to as a β″ “eye” is circled in yellow in (**a**). Precipitates in deformed specimens typically have regions (dashed) with reduced contrast/resolution along some of the edges.

Figure 3 presents filtered averages of smart-aligned^31^ HAADF STEM image stacks of precipitate cross-sections in specimens from different compression levels. The atomic columns of precipitates are resolved in both undeformed and deformed specimens, and the β″ eyes can be easily identified. This means that the precipitates do not change their overall structure after deformation. However, precipitates in deformed specimens usually exhibit blurred regions close to some of its edges where the β″ eyes cannot be identified. The appearance of these regions varies from precipitate to precipitate, and also with the focus of the electron probe.

### Study of the longitudinal precipitate structure

**Figure 4:**
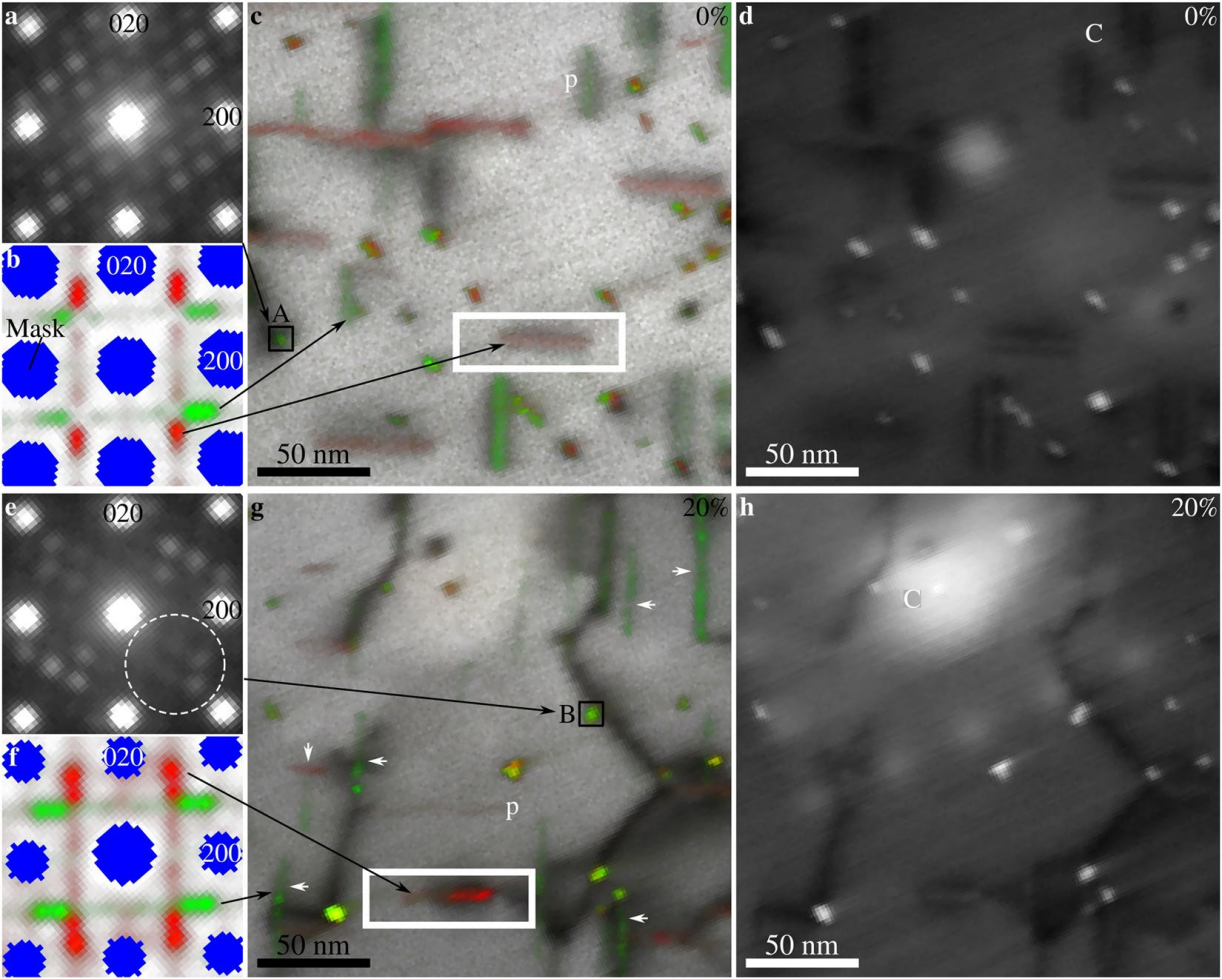
[001]_Al_ zone axis SPED results from undeformed (**a**–**d**) and 20% compressed (**e**–**h**) specimens. Single-pixel precession electron diffraction patterns of precipitate cross-sections marked “A” and “B” are shown in (**a**) and (**e**), normalised NMF factors from in-plane precipitates are shown in (**b**) and (**f**), VBF with overlaid normalised NMF loadings are shown in (**c**) and (**g**), while precipitate cross-section sensitive VADF images are shown in (**d**) and (**h**). NMF loadings also highlight some precipitate cross-sections and the VADF images should be used to separate precipitate segments and cross-sections. Green pixels in the factors and loadings are due to precipitates with $[010]$_β″_ $\Vert {[010]}_{{\rm{A}}{\rm{l}}}$ (vertical orientations), while red pixels are due to precipitates with $[010]$_β″_ $\Vert {[100]}_{{\rm{Al}}}$ (horizontal orientations). Precipitates in the deformed specimen appear segmented, some are marked by white arrows. Blue areas in the NMF factors correspond to reflections from the aluminium matrix that were masked out during the decomposition. The dashed circle in (**e**) marks weak precipitate reflections. The framed precipitates in (**c**) and (**g**) are studied in greater detail in Fig. 5. “C” marks amorphous contamination features formed during SPED alignment and preliminary SPED scans.

Figure 4 presents results from SPED experiments on specimens in undeformed and 20% compressed conditions. Only a small part of the data is cropped and presented here to make details more clear (see Supplementary Fig. S2 for the complete field of view). Precipitates in 5% and 10% compressed specimens show results similar to the 20% compressed specimens, and have been omitted for brevity (see the Supplementary Figs. S3–S6 for more detailed presentation of individual NMF factors and loadings for undeformed, 5%, 10%, and 20% compressed conditions, respectively). VBF and VADF images of the two conditions are shown, along with selected precession electron diffraction patterns and NMF results. There are two distinct orientations for each set of in-plane needle axis orientations. Green pixels show precipitates with their longitudinal axis aligned vertically ($[010]$_β″_ $\Vert {[010]}_{{\rm{A}}{\rm{l}}}$), while red pixels show precipitates aligned horizontally ($[010]$_β″_ $\Vert {[100]}_{{\rm{Al}}}$). NMF separates both variants for each orientation of the longitudinal axis. In undeformed specimens, the SPED results are as expected. Precipitates are surrounded by strain fields and appear straight with an even distribution of NMF loading intensity. The selected precession electron diffraction pattern of a precipitate cross-section shows that out-of-plane needles are in a [010]_β″_ zone axis (compare with the fast Fourier transform spectra insets in Fig. 2). After 20% compression, however, the precipitates scatter differently. Firstly, no strain field contrast is observed around in-plane precipitates. Secondly, the selected precession electron diffraction pattern indicates that scattering from precipitate cross-sections is suppressed, especially for some of the small scattering angles. Finally, the NMF loading intensities vary along the needles. This is clear from the loading maps, where the colouring appears segmented and irregular. Sometimes, this segmentation appears like a series of spots, and the VADF images must be used to establish whether a spot is due to a cross-section, or is indeed due to an in-plane needle.

**Figure 5:**
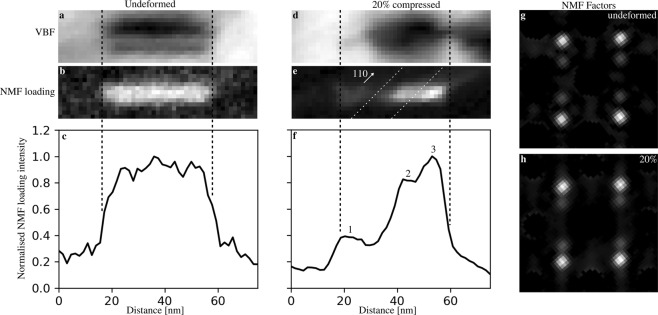
Close-up comparison between in-plane needles (marked by boxes in Figs. 1 and 4) in SPED scans of undeformed (**a**–**c** and **g**) and 20% compressed specimens (**d**–**f** and **h**). VBF images are shown in (**a**) and (**d**), while NMF loadings corresponding to the factors shown in (**g**) and (**h**) are shown in (**b**) and (**e**). Vertical sums (integrated profiles) of the NMF loadings are shown in (**c**) and (**f**). Note that these profiles do not correspond to single lines through the NMF loading maps. Three plateaus are identified in the profile of the needle in the 20% compressed specimen, labelled 1, 2, and 3. The interfaces between the regions in (**e**) that correspond to these plateaus are shown as dashed lines and are approximately along [110]_Al_. The NMF factors of the loadings are shown in (**g**) and (**h**).

Figure 5 shows a close-up comparison between a needle in undeformed and in 20% compressed condition. The needles have the same orientation and therefore, their most significant NMF factor is also similar. While the needle in the undeformed specimen shows a clear strain field in VBF, the needle in the 20% compressed specimen does not. Instead, this needle shows a dark contrast at one end, which is due to a dislocation that is pinned by the precipitate, as seen in Figs. 1d and 4g. The NMF loading intensities of the two needles are also different, being relatively constant in the undeformed specimen, whereas it varies along the needle in the 20% compressed specimen. There are three plateaus visible in the NMF loading profile of the needle in the deformed specimen, and these correspond to three regions with different intensity in the loading map. This indicates that the different parts of the same precipitate scatter electrons differently. The interfaces between these parts are also important. They are slanted with respect to the longitudinal direction of the needle and of significant width. The width of these interface regions indicates that the interface plane is inclined to the electron beam which travels along [001]_Al_. Hence, the interface between the different regions cannot be any of the {001}_Al_ planes. Instead, the ($1\bar{1}1$)_Al_ plane is more likely, as this will both have a trace along [110]_Al_ in the (001)_Al_ plane, and be inclined to the electron beam. Because this is one of the primary glide planes for dislocations in the matrix, it is likely that the regions visible in the NMF loading maps are related to shearing of the precipitate by dislocations gliding on these planes. Due to the slow decay of the phosphorous screen used to acquire the precession diffraction patterns, afterglow effects cause the NMF loading maps to smear out in the fast-scan direction (up towards the right-hand side corner of the maps in Fig. 5). Comparing the right-hand side of the NMF loading map shown in Fig. 5e with the slope seen in its vertical sum in Fig. 5f after ≈60 nm shows that afterglow effects are present, but that they are not a significant part of the NMF loading.

Finally, it should be mentioned that the variation of in-plane needle NMF loadings only means that the different regions scatter in different ways. Thus, the actual scattering may vary within the dark segments as well, as the NMF loadings only show that the scattering in these regions is different compared to the bright regions. This means that different parts of dark regions may, in principle, also scatter differently from each other. The intensity of the reflections in the precession electron diffraction patterns corresponding to in-plane needles is often too weak to be seen in the raw data, and it is therefore not possible to say anything about the actual differences between the dark and bright regions in the NMF loadings. It might therefore be that the total scattering in the dark regions is weaker, or that the dark regions scatter to other angles.

## Discussion

The HRTEM results show that the internal precipitate structure is affected by deformation. Because HAADF STEM images show that the precipitate interiors have the expected β″ structure, the blurry HRTEM images cannot be due to an overall structural change. This suggests that when β″ precipitates are sheared, the internal structure must change locally. Such local changes must be planar defects. Furthermore, this means that the shearing is incompatible with the precipitate structure, i.e. that the precipitates are sheared by matrix Burgers vectors. Hence, due to differences in precipitate and matrix structure, the planar defects cannot be anti-phase boundaries. In turn, this will complicate order strengthening, as this strengthening contribution traditionally assumes the formation of anti-phase boundaries^8^. Without knowing the structure of the planar defects, however, it is impossible to determine exactly how order strengthening is affected by this change. In addition, the shearing introduces steps at the precipitate-matrix interface, which increase the projected cross-sectional area of the precipitates and further blur the edges of precipitates in HRTEM. These steps can be inferred indirectly from the more rounded and larger cross-sections of out-of-plane precipitates observed in HRTEM images of deformed specimens (see Supplementary Fig. S1). However, this inference is strengthened by the fact that steps are a natural consequence of the shearing process. Whether the shearing is achieved by a gliding dislocation through the precipitate or by a collective shift of the precipitate structure is not known however, and the step size is uncertain.

**Figure 6:**
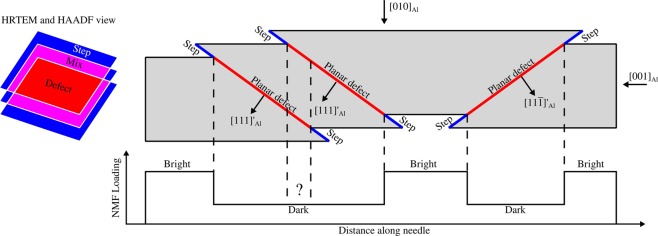
Schematic illustration of a [100]_Al_ section of a sheared β″ precipitate and how deformation induced defects will appear in HRTEM, HAADF STEM and SPED. The precipitates are sheared on {111}_Al_ planes (marked by the projection of their 〈111〉′_Al_ plane normals), and the shearing planes become planar defects because these are not slip planes in the β″ phase. Each shearing event also creates steps on the precipitate-matrix interface. Traces of the planar defects and steps are shown in red and blue, respectively. The cross-sectional view to the left is a projection of the needle along [001]_Al_, and shows the defects and steps in projection, with overlap between planar defects and steps shown as magenta regions. The SPED NMF loading intensity variation of in-plane needles is shown schematically as well and is explained as the projection of the planar defects along [010]_Al_. Whether the projection of the steps influence scattering is not known.

Figure 6 summarises our interpretations of the results. This figure illustrates that the inferred steps and the observed planar defects induced by the shearing may overlap when projected along the needle longitudinal axis. When a sheared precipitate is observed along the needle lengths using HRTEM or HAADF STEM, the images will contain regions that are a mix of β″ and matrix structures (from the steps) and disrupted structures (from the planar defects). The figure also shows schematically how the defects overlap when projected perpendicularly to the needle longitudinal direction, as is the case in SPED (see Figs. 4 and 5). The NMF results from SPED show that in-plane needles have small regions that scatter as expected, and other regions that scatter differently. Our hypothesis is that the planar defects disrupt scattering enough to make the loadings of the NMF factors of the in-plane needles considerably weaker. This is based on the fact that the atomic disruptions introduced by a planar defect likely will affect one or two β″ eyes above and below it, and that this region makes out a significant part of the precipitate as the beam travels through its transverse direction. In addition, the signal from in-plane needles is originally very weak in undeformed specimens, and any reduction in this signal will influence the NMF loadings. Hence, wherever the beam intersects one of these planar defects, the resulting precession electron diffraction pattern will contain less intensity in corresponding diffraction spots. The width of the interface between the NMF loading plateaus in the needle in Fig. 5 indicates that the planar defects are observed at an angle, and that they must lie on any other plane than {001}_Al_. This is consistent with shearing on {111}_Al_ matrix glide planes, and we therefore propose that shearing of β″ precipitates does not require cross-slip to e.g. the {001}_Al_ planes. In addition, due to the interfaces and the large and continuous dark regions (the plateaus in Fig. 5), we suggest that several closely spaced planar defects overlap when needles are observed along 〈100〉_Al_ and perpendicular to their longitudinal axis, as illustrated in Fig. 6. Repeated shearing events in the same plane seem unlikely, as this would lead to relatively narrow dark regions in the NMF loading maps and bigger steps on the needle interfaces. However, this has not been possible to confirm directly. If this interpretation is correct, it will mean that the loss of precipitate strength due to a reduction in cross-sectional area in the shearing plane is compensated by a local increase in hardening contributions in the shearing plane. This local hardening would then cause dislocations gliding in the same plane to e.g. cross-slip to nearby planes before shearing the precipitate and introduce new planar defects. As more and more planes become sheared, dislocations must cross-slip further and further in order to find an undisturbed part of the precipitate to shear through. This hypothesis agrees with observations made by Poole *et al*.^17^ on an Al-Mg-Si-Cu alloy and is similar to processes proposed for the Al-Cu-Li alloy studied by Deschamps *et al*.^9^. The relaxation of precipitate strain fields observed by bright field TEM will likely also play a role when considering repeated shearing of precipitates. The source of this relaxation is unknown, and it might itself be a result of repeated shear on nearby planes, but its most probable effect is that coherency strengthening becomes less important after some shearing events have occurred.

HAADF STEM shows that the atomic structure of the precipitate interiors is unchanged by deformation. How the blurred regions observed in the HAADF STEM results fit together with the planar defects and steps revealed by HRTEM and SPED is challenging to explain. First of all, a sheared precipitate should contain three different regions when observed along its longitudinal axis; an interface step on one side where the beam first travels through the matrix and then through the precipitate, an internal region where the beam travels across the planar defect, and finally a second interface step on the other side where the beam first travels through the precipitate and then through the matrix. The HAADF STEM images in Fig. 3 do indeed show three separate regions in precipitate cross-sections in deformed specimens, namely two blurry endge regions (marked by dashed lines) and one larger well-resolved region in-between. It therefore seems likely that the blurry regions in the STEM images are due to the steps, and the well-resolved region corresponds to the area with overlapping β″ structures and a planar defect somewhere in the thickness direction. However, this raises the question: why does the internal structure of sheared precipitates appear blurry in HRTEM, but well-resolved in STEM? If the atomic columns of the precipitate are well-aligned above and below the shearing plane, the STEM images should show well-resolved β″ eyes. However, this would mean that the precipitates are sheared in a manner compatible with their structure. This is at odds with both the SPED and the HRTEM results, however, and therefore does not offer a satisfactory explanation. Hence, it is more likely that the difference between the HRTEM and HAADF STEM results originates from the image forming mechanisms. A possible explanation in this regard is that only parts of the precipitate contribute to the STEM image, as STEM is most sensitive to the specimen closest to the entrance surface. However, if this was the case, the steps should not be blurry either. The fact that the steps remain blurry and detectable indicates that the shearing plane lies close enough to the entrance surface to contribute significantly to the image. The contrast in the STEM images therefore cannot be explained solely by the sequential nature of the STEM image forming mechanisms. Another explanation is that the beam channelling conditions might be very different in the different regions. However, to address this issue, detailed STEM image simulations coupled with accurate structural models of sheared precipitates are necessary, but out of scope of the current study.

Our results support observations and experiments done by Poole *et al*.^17^ and Ryen *et al*.^16^. Poole *et al*. observed macroscopically that β″ precipitates must be sheared, but could not observe this directly. This is not surprising, given the detailed study required for such observations. Meanwhile, the rotations of the precipitates longitudinal axis observed by Ryen *et al*. can be explained by several shearing events of the needles in a preferred direction. In addition, the planar defects observed in the present work provide information of the shearing process on the sub-nanometre level, which may be used to refine simulations that previously only accounted for the precipitates elastic strain fields^14^. However, some important details are still missing, but further studies based on atomistic simulations of sheared structures coupled with TEM image simulations may provide more detailed insight in the future.

## Conclusion

We conclude that β″ precipitates in peak hardness Al-Mg-Si alloys are sheared several times by matrix dislocations gliding on {111}_Al_. These shearing events are not compatible with the precipitate phase, i.e. the relative translations involved in the events are not Burgers vectors of the precipitate, and results in planar defects within the precipitates. The planar defects are not anti-phase boundaries, but their exact atomic structure is unknown. They are inclined to 〈001〉_Al_ directions and appear to be closely spaced, but not co-planar, suggesting that dislocations avoid shearing a precipitate in the same plane. These conclusions are important for further development of models of strength, work hardening, and fracture of alloys containing these precipitates.

## Methods and Materials

### Material

The AA6060 aluminium alloy used in this study has been investigated in several other studies, and the reader is referred to these previous works for details, while a short summary is presented here^32–34^. The alloy contained 0.422 wt% Si and 0.468 wt% Mg, and was extruded as a 10 mm thick profile measuring 83 mm in width. Cylindrical compression specimens (13 mm in length and 9 mm in diameter) were machined with their longitudinal direction along the transverse direction (TD) of the profile. These compression specimens were then solution heat-treated at 540 °C for 15 min before being quenched in water. They were subsequently naturally aged for 15 min at room temperature, and finally artificially aged to peak hardness at 185 °C for 5 hours. After heat-treatment, the average precipitate length, cross-section, number density, and volume fraction measured by TEM were $\bar{l}$ = 40 ± 1 nm, $\bar{\sigma }$ = 19.1 ± 0.8 nm^2^, $\bar{\rho }$ = 5556 ± 629#/μm^3^, and ${\bar{V}}_{f}$ = 0.42 ± 0.05%, respectively^34^. All of the observed precipitates were consistent with the β″ phase, except for relatively few inhomogeneously nucleated precipitates that were of an over-aged character. The material has an equiaxed grain structure with a strong cube texture and minor Goss components^32,33^.

### Compression tests

Three heat-treated compression specimens were subjected to uniaxial compression at a strain rate of 2 × 10^−4^ s^−1^ in a universal testing machine to a final engineering strain of 5%, 10% and 20%, respectively. *Dow Corning Molykote G-n Metal Assembly Paste* was used to reduce the friction at the contact surfaces between the specimen and the platens, resulting in barrelling less than 6%.

### Thin foil preparation

TEM thin foils were made by sectioning the compression specimens along the extrusion plane so that the global compression axis lies in the thin foil plane. Sections close to the specimen centres were mechanically polished down to ≈300 μm before standard 3 mm disks were punched out. The disks were further polished down to ≈100 μm before they were electropolished with a standard electrolyte (a mixture of 1/3HNO_3_ and 2/3CH_3_OH) in a *Struers TenuPol 5* twin-jet electropolisher. The electrolyte was kept at −25 ± 5 °C, and a voltage of 20 V was applied. The thin foils were plasma cleaned in a *Fischinone 1020 Plasma Cleaner* for 5 min before the TEM investigations. It should be noted that the preparation of TEM thin foils and the investigation itself were conducted three years after the completion of the compression tests. In the meantime, the specimens were stored at room temperature.

### Microscopy

A *JEOL JEM2100F* operating at 200 kV was used to perform HRTEM and SPED. A *Gatan 2k UltraScan CCD* was used for the HRTEM studies. More than 50 precipitates for each condition were imaged in HRTEM, and representative images reflecting this variation were chosen for this paper. SPED was done by the *NanoMEGAS DigiSTAR* system, with a precession angle of 1° and precession frequency of 100 Hz^22,24^. The nominal probe size was 1 nm, and a step size of 1.52 nm was used to scan the specimen. Typical scan sizes were 400 × 400 pixels (608 × 608 nm^2^). An external *Allied StingRay* CCD camera binned to 144 × 144 pixels with 8-bit depth was used to acquire images of the phosphorous screen (showing the precession electron diffraction patterns) with an exposure time of 40 ms. The exposure time and typical scan size resulted in acquisition times of approximately two hours.

A double-corrected cold-FEG *JEOL ARM200F* operating at 200 kV was used to perform HAADF STEM imaging of precipitate cross-sections to complement the HRTEM studies. The images were acquired using the *Smart Align* plugin to *Gatan Digital Micrograph*, and both rigid and non-rigid registration were used to produce aligned stacks of images formed with a collection angle of 48–206 mrad^31^. HAADF images presented in this work are averages through these aligned stacks. The scans were conducted with a pixel dwell time of 2 μs and with pixel sizes <8 pm. Typically, 10 or 12 frames were acquired for each image stack. Conventional HAADF STEM images of each area were also acquired to validate the smart aligned images.

### Analysis

*ImageJ* was used to measure the cross-sectional area of precipitates from HRTEM images. Only precipitates in undeformed and 20% compressed specimens were measured. There are significant uncertainties and error sources connected to these measurements, the largest being difficulties in determining the edges of precipitate cross-sections in deformed specimens. The relative error in cross-sectional area measurements for precipitates in the deformed state is estimated to be approximately 10%. Only precipitates in a single grain were measured. The average cross-sectional area measured in undeformed alloys matches perfectly that of a previous study, which suggests that the variation in precipitation in different grains is limited in this material^34^.

HAADF STEM images were filtered in Fourier space by applying a mask with radius 8.38 1/nm ≈1.2 · *d*_220_, where *d*_220_ is the reciprocal spacing of the aluminium (220)_Al_ planes. The edge of the mask had a Gaussian shape with a half-width of 10% of the mask radius. Intensities of all TEM, HRTEM, HAADF STEM, and virtual SPED images in this paper have been limited to the 0.01 and 0.99 percentiles of the data to enhance contrast.

The open-source Python packages *HyperSpy* and *PyXem* were used to create VBF and VADF images of the SPED data^35,36^. These virtual images were made by assigning pixel intensities based on the integrated intensity within certain regions of the corresponding precession electron diffraction patterns. For VBF images, this region was 0–4.91 · 10^−4^ mrad (slightly larger than the size of the direct beam), while for VADF images, intensities within the virtual annulus of 4.83 · 10^−4^–1.29 · 10^−2^ mrad (from the edge of the direct beam and up to the edge of the {002}_Al_ reflections) were used.

The same python packages were used to perform unsupervised NMF machine learning of the SPED data^21^. In the current work, the NMF algorithm was used to separate the data into 100 linearly independent factors and corresponding loadings. The factors and loadings corresponding to in-plane needles are of special interest and were extracted from the NMF results by manual examination. This was done by comparing the factors to simulated kinematic selected area electron diffraction patterns of in-plane needles of the β″ phase, and by considering the corresponding loadings. Electrons propagating perpendicularly to the needle longitudinal directions may scatter from planes with a relatively large excitation error because of the thin needle dimensions. Hence, needles of different phases can scatter quite similarly when observed edge-on. Therefore, several different phases contribute to the in-plane needle NMF factors and loadings. Similarly, these factors will also contain some signals that overlap with the signal from precipitate cross-sections, and the loading maps will therefore also show precipitate cross-sections. VADF images can be used to identify the cross-sections, and the NMF loading maps should be considered in parallel with these. Vertical integrated profiles of NMF loadings of select precipitates (see Fig. 5) were made by rotating the NMF loadings to align the precipitates horizontally, and sum the resulting interpolated pixel values of the rotated loading along the image vertical axis.

### Distortions in SPED

Because the acquisition time of SPED scans can be quite long (~hours), specimen drift will distort the scans and cause a square scan grid to become non-square on the specimen. This distortion is important to have in mind when considering the SPED results, as it affects angles between objects in virtual images. Specimen drift is unavoidable but can vary in severity. Therefore, conventional TEM images have been used to validate the SPED scans and to make sure that the scan distortions are not too severe. In principle, the SPED scans can be made to fit the TEM images using affine transformations, but such processing has not been done in the current work. In addition, the precession electron diffraction pattern is formed on a tilted screen, and the external camera is also tilted relative to the screen. Hence, the resulting precession electron diffraction patterns are also distorted. However, this distortion is of little consequence to the present study, as the interest is in relative orientations and qualitative considerations. Finally, afterglow effects, due to the slow and non-linear phosphorous screen, will smear features along the fast-scan direction during SPED acquisition and lead to artificial overlap between phases. This experimental artefact limits the quantitative possibilities of SPED and warrants special care when interpreting e.g. NMF results.

## Supplementary information

Supplementary Information

## Data Availability

The datasets generated during and/or analysed during the current study are available in the Zenodo repository, 10.5281/zenodo.2652906^37^. This repository contains many more TEM, HRTEM, and STEM images than shown here, in addition to the smart-align image stacks.
