# Supplementary material for: Nano-scale characterisation of sheared β” precipitates in a deformed Al-Mg-Si alloy
Source: Sci Rep. 2019 Nov 25;9:17446. doi: 10.1038/s41598-019-53772-4 (PMC6877512; doi:10.1038/s41598-019-53772-4)
Supplement: Supplementary file 1 — Supplementary Information [file 41598_2019_53772_MOESM1_ESM.pdf]

Supplementary Information

Nano-scale characterisation of sheared β″ precipitates in a deformed Al-Mg-Si alloy

Emil Christiansen[1,2,*], Calin Daniel Marioara[1,3], Bjørn Holmedal[1,4], Odd Sture Hopperstad[1,5], Randi Holmestad[1,2].

[1]Centre for Advanced Structural Analysis (CASA), NTNU – Norwegian University of Science and Technology, Trondheim, N-7491, Norway.
[2]Department of Physics, Faculty of Natural Sciences, NTNU, Trondheim, N-7491, Norway.
[3]Materials and Nanotechnology, SINTEF Industry, Trondheim, N-7465, Norway.
[4]Department of Materials Science and Engineering, Faculty of Natural Sciences, NTNU, Trondheim, N-7491, Norway.
[5]Department of Structural Engineering, Faculty of Engineering, NTNU, Trondheim, N-7491, Norway.
[*]Corresponding author, Emil Christiansen, emil.christiansen@ntnu.no.

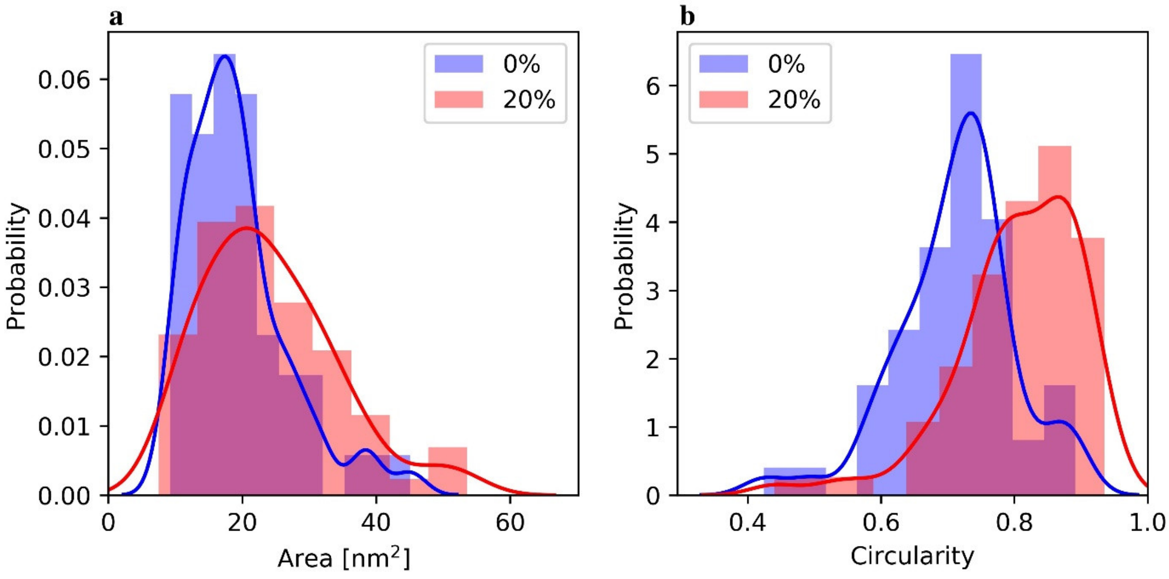

Supplementary Figure S1: Precipitate cross-sectional area (**a**) and circularity (**b**) in AA6060 before (blue) and after 20% compressive engineering strain (red). Kernel density estimates are included as a guide. The circularity is the ratio between the measured area and the area of an equivalent circle with the circumference identical to the measured circumference of the precipitate cross-section. Precipitate cross-sections tend to become larger and more varied after deformation. They also tend to have circularity values closer to unity, indicating that they become more equiaxed and circular compared to the undeformed case.

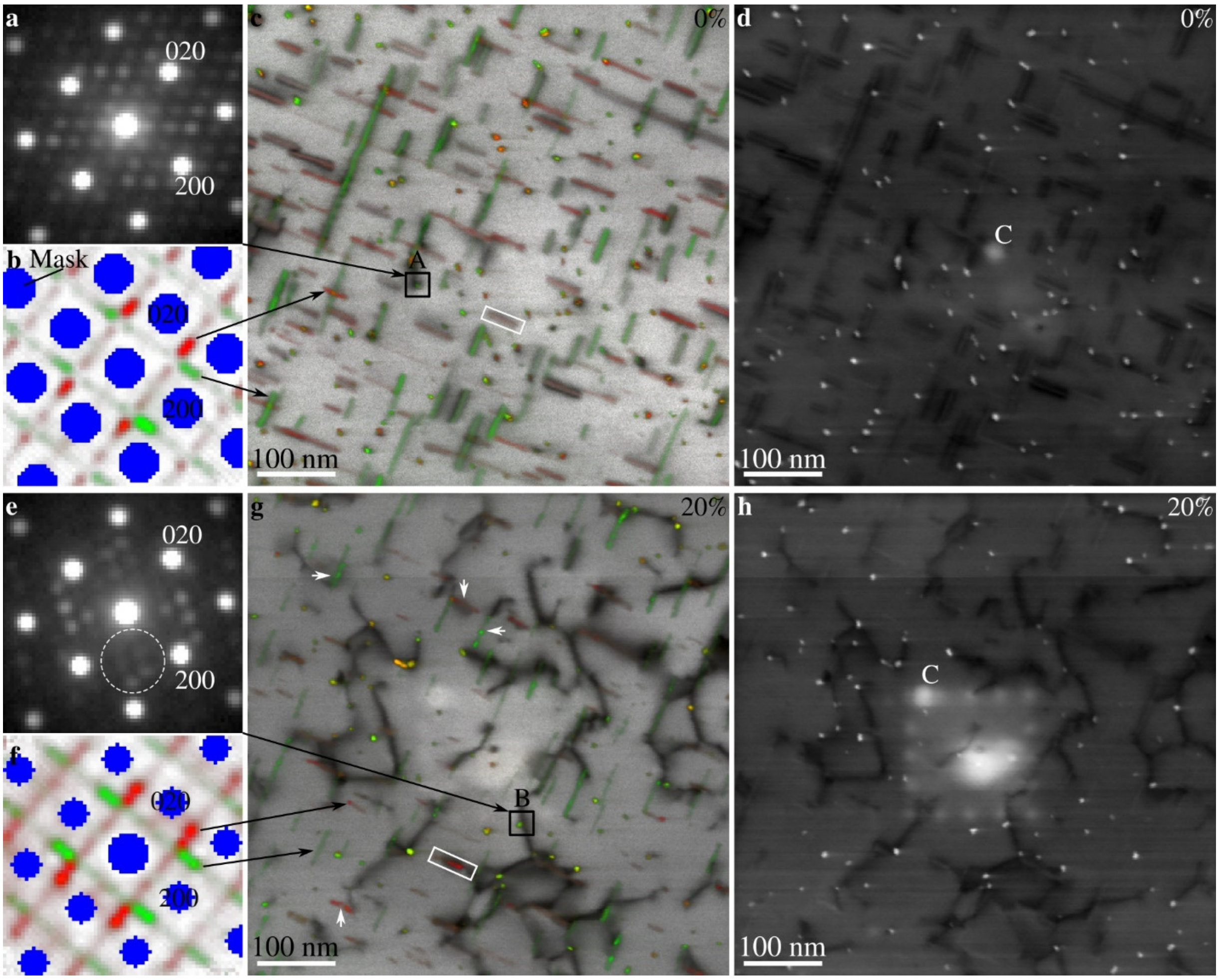

Supplementary Figure S2: Complete $[001]_{Al}$ zone axis SPED results from undeformed (**a**-**d**) and 20% compressed (**e**-**h**) specimens, showing the complete region from which the results shown in Fig. 4 are taken from. Single-pixel precession electron diffraction patterns of precipitate cross-sections marked "A" and "B" are shown in (**a**) and (**e**), normalised NMF factors from in-plane precipitates are shown in (**b**) and (**f**), VBF with overlaid normalised NMF loadings are shown in (**c**) and (**g**), while precipitate cross-section sensitive VADF images are shown in (**d**) and (**h**). NMF loadings also highlight some precipitate cross-sections and the VADF images should be used to separate precipitate segments and cross-sections. Green pixels in the factors and loadings are due to precipitates with $[010]_{\beta''} \parallel [010]_{Al}$ (vertical orientations), while red pixels are due to precipitates with $[010]_{\beta''} \parallel [100]_{Al}$ (horizontal orientations). Precipitates in the deformed specimen appear segmented, some are marked by white arrows. Blue areas in the NMF factors correspond to reflections from the aluminium matrix that were masked out during the decomposition. The dashed circle in (**e**) marks weak precipitate reflections. The framed precipitates in (**c**) and (**g**) are studied in greater detail in Fig. 5. "C" marks amorphous contamination features formed during SPED alignment and preliminary SPED scans.

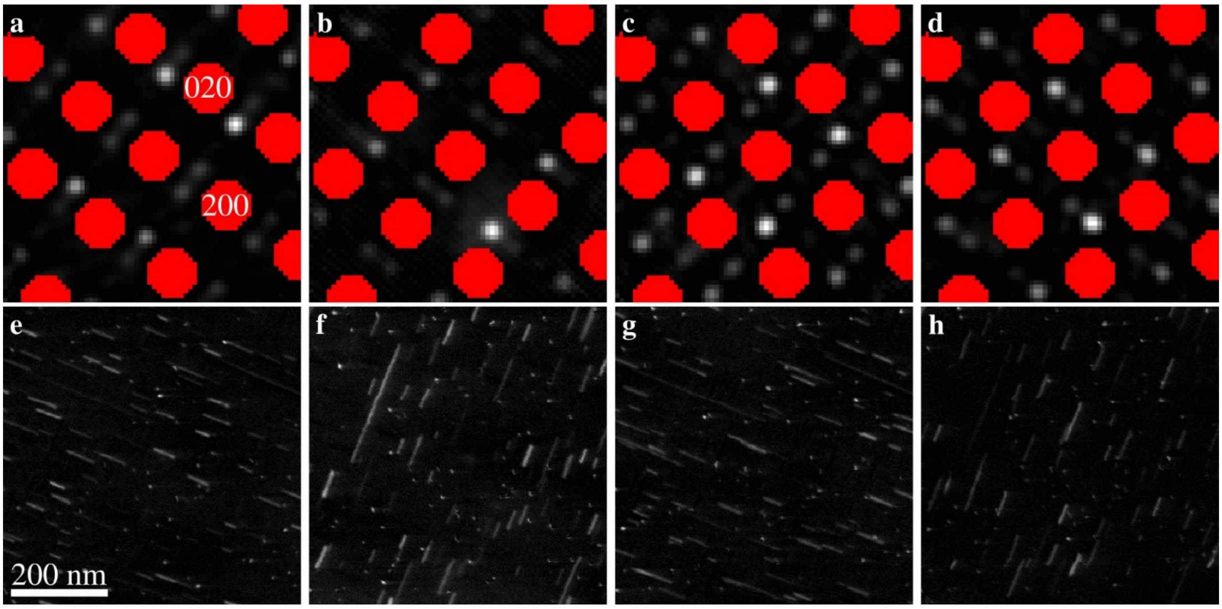

Supplementary Figure S3: Non-negative matrix factorisation factors (**a**-**d**) and loadings (**e**-**h**) related to in-plane β" precipitates learned from scanning precession electron diffraction data of AA6060. (**a**,**e**), (**b**,**f**), (**c**,**g**), and (**d**,**h**) are different factor-loading pairs that correspond to different orientations of the β" phase. Red areas in the factors are regions that were masked out during the factorisation to enhance sensitivity to the signals from precipitates.

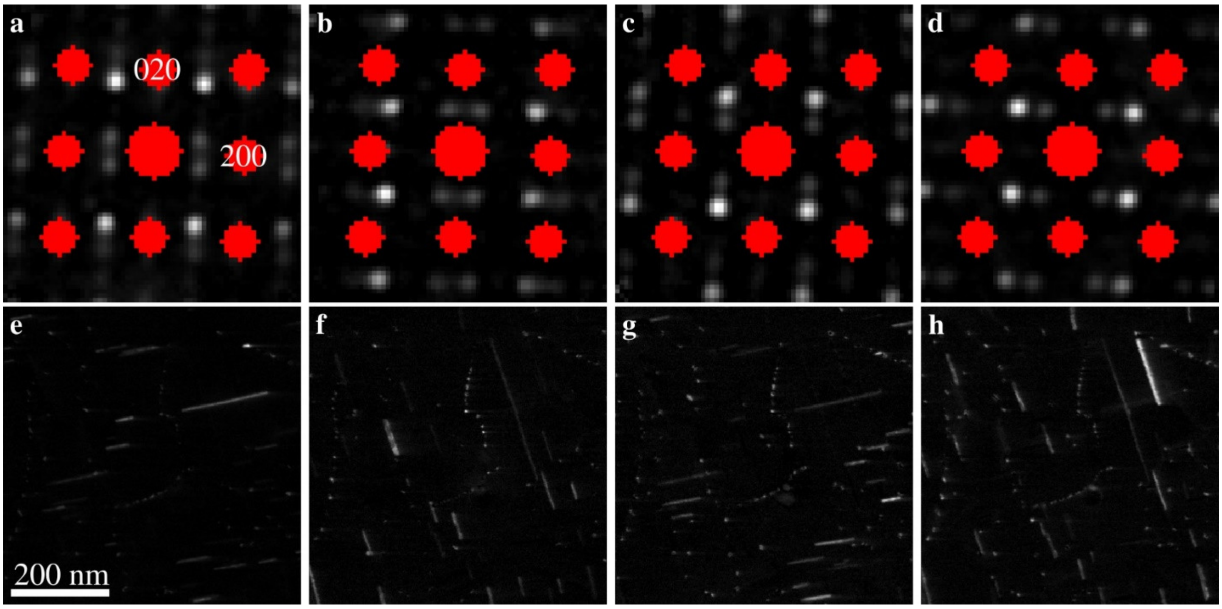

Supplementary Figure S4: Non-negative matrix factorisation factors (**a**-**d**) and loadings (**e**-**h**) related to in-plane β" precipitates learned from scanning precession electron diffraction data of AA6060 compressed to 5% engineering strain. (**a**,**e**), (**b**,**f**), (**c**,**g**), and (**d**,**h**) are different factor-loading pairs that correspond to different orientations of the β" phase. Red areas in the factors are regions that were masked out during the factorisation to enhance sensitivity to the signals from precipitates.

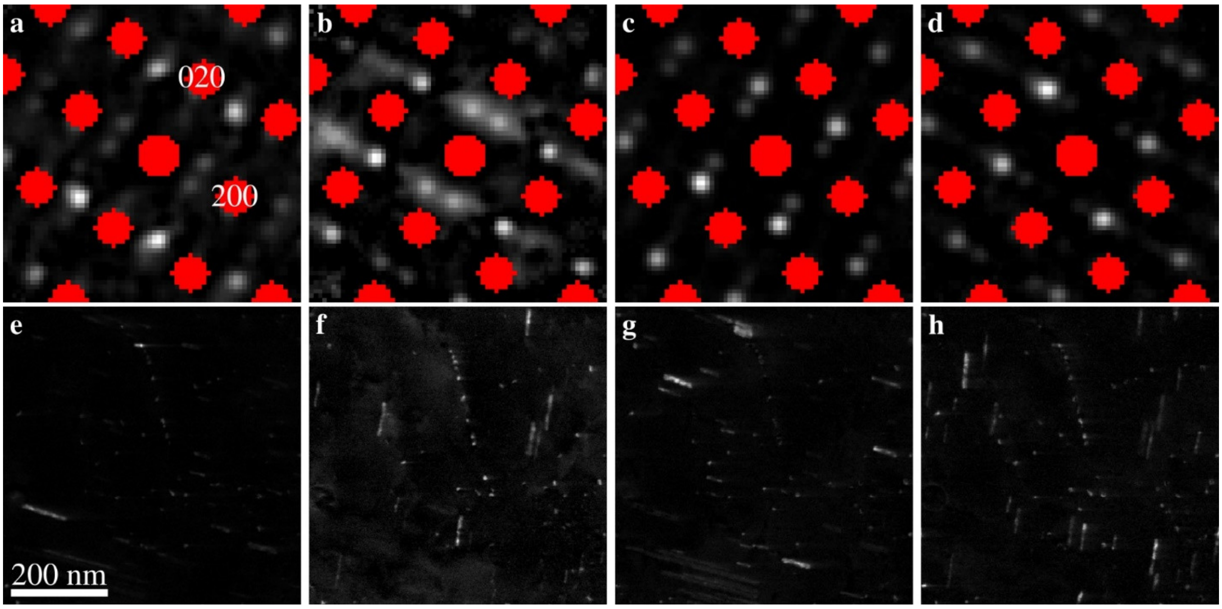

Supplementary Figure S5: Non-negative matrix factorisation factors (**a**-**d**) and loadings (**e**-**h**) related to in-plane β" precipitates learned from scanning precession electron diffraction data of AA6060 compressed to 10% engineering strain. (**a**,**e**), (**b**,**f**), (**c**,**g**), and (**d**,**h**) are different factor-loading pairs that correspond to different orientations of the β" phase. Red areas in the factors are regions that were masked out during the factorisation to enhance sensitivity to the signals from precipitates.

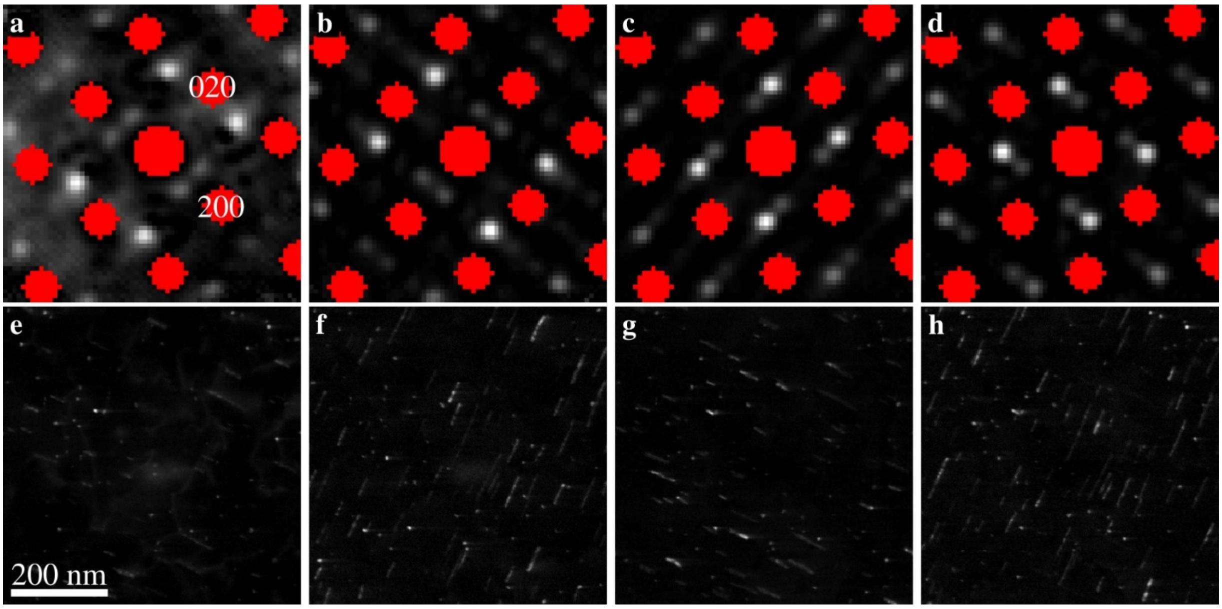

Supplementary Figure S6: Non-negative matrix factorisation factors (a-d) and loadings (**e**-**h**) related to in-plane β" precipitates learned from scanning precession electron diffraction data of AA6060 compressed to 20% engineering strain. (**a**,**e**), (**b**,**f**), (**c**,**g**), and (**d**,**h**) are different factor-loading pairs that correspond to different orientations of the β" phase. Red areas in the factors are regions that were masked out during the factorisation to enhance sensitivity to the signals from precipitates.
